# Synergistic Effect of Liraglutide and Strength–Endurance Exercise Training on Hepatic Oxidative Stress and Lipid Metabolism in Middle-Aged Male Rats

**DOI:** 10.3390/antiox14121492

**Published:** 2025-12-12

**Authors:** Dragana Vlahović, Svetlana Trifunović, Slavica Borković-Mitić, Slađan Pavlović, Ivona Gizdović, Dieter Lütjohann, Branko Filipović, Ljiljana Marina, Branka Šošić-Jurjević

**Affiliations:** 1 Institute for Biological Research “Siniša Stanković”—National Institute of the Republic of Serbia, University of Belgrade, Bulevar despota Stefana 142, 11108 Belgrade, Serbia; dragana.vlahovic@ibiss.bg.ac.rs (D.V.); lanat@ibiss.bg.ac.rs (S.T.); borkos@ibiss.bg.ac.rs (S.B.-M.); sladjan@ibiss.bg.ac.rs (S.P.); ivona.gizdovic@ibiss.bg.ac.rs (I.G.); brankof@ibiss.bg.ac.rs (B.F.); 2 Institute of Clinical Chemistry and Clinical Pharmacology, University Hospital Bonn, Venusberg–Campus 1, 53127 Bonn, Germany; dieter.luetjohann@ukb.uni-bonn.de; 3 Center for Infertility and Endocrinology of Gender, University Clinical Center of Serbia, Faculty of Medicine, University of Belgrade, 11000 Belgrade, Serbia; ljiljana.marina@med.bg.ac.rs

**Keywords:** GLP-1RA, exercise, middle–age, rats, liver, oxidative stress, lipid metabolism

## Abstract

Glucagon-like peptide-1 receptor agonists and lifestyle interventions effectively treat overt obesity, but the benefits/risks of their combined early intervention during middle age remain unclear. This study investigated whether submaximal-dose liraglutide combined with strength–endurance training improves metabolic and liver health, focusing on hepatic oxidative stress and lipid metabolism. Male Wistar rats (16 months old) received liraglutide (L; 0.186 mg/kg/day, s.c.), training (ladder climbing with weights, 3 times/week), both (L+E) or saline for control middle-aged (C) and young adults (CY; 3–4 months old) for 7 weeks (n = 8/group). Middle-aged rats exhibited age-related changes including higher body and visceral fat, increased hepatic and serum cholesterol, hepatic ALT and glutathione imbalance, and decreased soleus muscle (*p* < 0.05, vs. CY). Exercise increased hepatic glycogen and oxidative stress markers and downregulated lipogenic genes, consistent with liver adaptation to training. L+E synergistically reduced body and visceral fat, hepatic and serum triglycerides, and the triglyceride–glucose index, while reducing oxidative stress (*p* < 0.05 vs. E, C) and lipogenic gene expression (*p* < 0.05 vs. C), without affecting pancreas histopathology and function parameters, muscle mass or exercise load volume. In conclusion, submaximal liraglutide safely synergized with training to enhance metabolic health, improve hepatic redox balance and triglyceride metabolism in middle-aged rats, without mitigating cholesterol rise.

## 1. Introduction

The escalating global prevalence of obesity, which is nowadays considered as chronic progressive metabolic disease closely linked to accelerated aging [1], underscores the need for early intervention strategies. The liver plays a central role in glucose and lipid metabolism, hormone regulation and degradation, and insulin actions—processes critical for energy homeostasis and healthy aging [2]. With advancing age, hepatic function declines, a process intensified by hormonal changes and a reduced capacity to counteract oxidative stress, leading to metabolic alterations that favor the conversion of excess glucose into fatty acids and lipids [2]. If not prevented or reversed, these changes may progress to overt liver and cardiometabolic diseases [3,4].

Glucagon-like peptide-1 receptor agonists (GLP-1RAs) have emerged as effective treatments for obesity, achieving 15–25% weight loss while improving glycemic control, cardiovascular outcomes, and hepatic metabolism [5]. Although GLP-1 acts through its receptor, the presence and functional relevance of hepatic GLP-1R remain debated [6,7]. Nevertheless, animal and clinical studies consistently show that GLP-1RAs reduce hepatic steatosis and inflammation [8]. Concerns persist, however, regarding skeletal muscle loss with potent agents such as semaglutide [9,10]. Liraglutide, though less potent in weight reduction, appears to preserve lean mass better and may therefore represent a safer therapeutic option for midlife and older adults [11,12].

Exercise remains a cornerstone of metabolic health, as it independently improves glucose regulation, cardiovascular performance, and hepatic integrity [13,14]. Resistance training, which combines strength and endurance components, is particularly effective for enhancing body composition, muscle metabolism, and overall fitness in middle-aged adults [15]. Recent findings suggest that combining GLP-1RAs with structured exercise may boost metabolic benefits while preserving muscle mass [16]. In rodents, ladder climbing is a validated model of combined resistance training, promoting both strength and endurance through progressively loaded climbs with brief recovery periods between repetitions until voluntary fatigue.

Middle age represents a critical transitional period during which both rodents and humans begin to exhibit subtle metabolic impairments, including overweight or obesity, increased visceral adiposity, and dysregulated glucose and/or lipid metabolism [17,18]. This early stage might offer a critical window for preventative interventions, yet current strategies are often initiated only after significant disease progression. While GLP-1 receptor agonists are widely used clinically [19], their efficacy as early metabolic interventions—before severe obesity metabolic disease develops—particularly when combined with lifestyle-based therapies, is largely unexplored. Results from preclinical studies, employing maximal or supramaximal liraglutide doses, show synergistic benefits with exercise in improving metabolic and vascular oxidative stress in diet-induced obesity models [20], also demonstrating effectiveness against hepatic steatosis and oxidative stress in metabolic-associated fatty liver disease models [8].

This study aimed to determine whether a submaximal dose of liraglutide—chosen for its pharmacological relevance in obesity treatment—synergizes with strength–endurance exercise training to improve metabolic and hepatic health. Liraglutide was selected as a less potent GLP-1 receptor agonist to minimize the risk of adverse effects and skeletal muscle loss. To address this aim, we examined changes in physiological and systemic metabolic parameters, pancreas and liver histology, key markers of hepatic oxidative stress, expression of lipid metabolism genes, and hepatic cholesterol and triglyceride concentrations in middle-aged male Wistar rats. To justify our model of mild spontaneous obesity and early metabolic dysfunction, we first compared physiological, metabolic, and hepatic features between young adult and middle-aged control animals. Early metabolic shifts are often modest and reflect compensatory adjustments, but because adaptive reserves and homeostatic robustness wane with age, comparing treatments against both age-matched and young adult controls is essential to reveal whether interventions restore function or provoke adverse responses.

## 2. Materials and Methods

### 2.1. Animals and Diets

A total of forty adult male Wistar rats were used in this study, including young adult (3–4 months old) and middle-aged rats (16 months old). The animals were bred and housed in the Unit for Experimental Animals at the Institute for Biological Research “Siniša Stanković”—National Institute of the Republic of Serbia. The animals were housed under controlled lighting (12 h light–12 h dark) and temperature (21 ± 2 °C) conditions with ad libitum access to food and water. The animals were fed a standard grain-based pellet diet (IG-Z-00117, Gebi d.o.o., Čantavir, Serbia), which comprised 86.5% dry matter and provided 1405 kJ (333 kcal) of energy per 100 g. The nutrient composition included 20.3% protein, 3.2% fat, 49% carbohydrates—of which 2.6% were sugars—12.9% fiber, and 8% ash. Rats were housed in groups of two per cage for the majority of the experiment. To enable accurate measurement of individual food intake and to minimize stress, animals were individually housed for 48 h at the beginning of the experiment and again for 48 h, four days before the sacrifice.

### 2.2. Ethical Approval

All procedures involving the animals were complied with Directive 2010/63/EU on the protection of animals used for scientific purposes. The ethical approval was obtained from the Veterinary Directorate of the Ministry of Agriculture, Forestry and Water Management of the Republic of Serbia on 30 January 2024, under the reference number 000169446 2024 14841 002 001 000 001. The approved sample size was calculated using G*Power 3.1 software with a type I error rate of α = 0.05, statistical power of 0.95, effect size (f) of 0.75, and a two-tailed alternative hypothesis (Supplementary Figure S1). Based on these parameters, the analysis indicated that a minimum of 8 rats per group was required. The experiments were performed following the guidelines of ARRIVE (Animal Research: Reporting In Vivo Experiments) and the guidelines on the principles of regulatory acceptance of 3R (replacement, reduction, refinement) testing approaches.

### 2.3. Study Design

**Figure 1:**
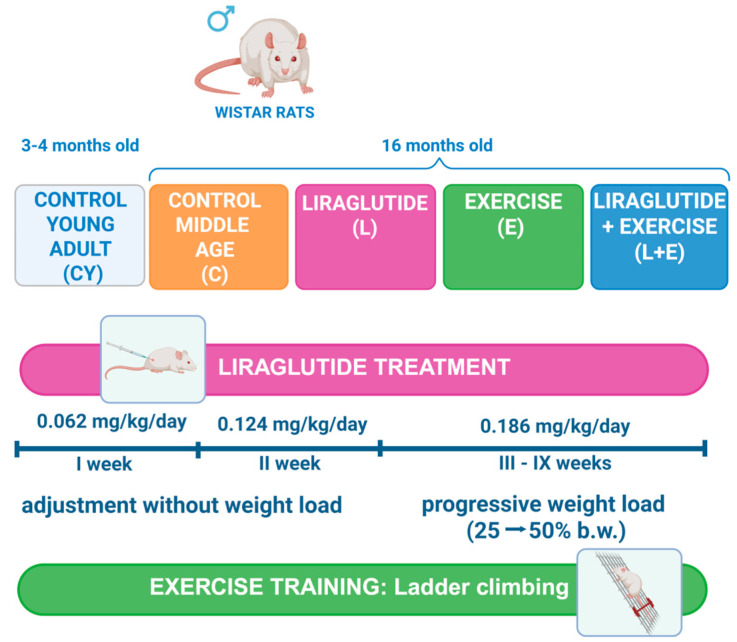
Schematic of the experimental design. Male Wistar rats were randomly assigned to groups (n = 8/group)—young adult (CY, 3–4 months old) and middle-aged (16 months old) groups—subdivided into control (C), liraglutide treatment (L), exercise (E), and combined liraglutide plus exercise (L+E). Rats in the liraglutide-treated groups (L and L+E) received subcutaneous injections of 0.3 mL liraglutide (Saxenda^®^, Novo Nordisk A/S, Bagsværd, Denmark) at a dose of 0.186 mg/kg body mass (b.m.) daily, dissolved in normal saline. The treatment started at a dose of 0.062 mg/kg/day and was increased weekly until reaching the final dose of 0.186 mg/kg/day, which was maintained for 7 weeks. The control (C and CY) and exercise (E) groups received an equal volume (0.3 mL) of the saline alone. The exercise training protocol included a two-week adaptation (climbing without weights), followed by 7 weeks of strength–endurance training involving ladder climbing (typically 4–5 times, 1 min rest, until failure) with progressive weight load (25–50% of body weight). Created in BioRender. Gizdovic, I. (2025) https://BioRender.com/31io6u5.

The experimental design is summarized in Figure 1.

The 16-month-old male rats were randomly assigned to four experimental groups (n = 8/group): liraglutide (L), exercise (E), liraglutide plus exercise (L+E), and control (C). The L and L+E groups received a daily subcutaneous injection of liraglutide (Saxenda^®^, Novo Nordisk A/S, Bagsværd, Denmark) at a dose of 0.186 mg/kg body mass, dissolved in normal saline (final volume of 0.3 mL). The C and E groups, as well as the control young adult group (CY: n = 8/group), received an equivalent volume of vehicle alone for nine weeks. All injections were administered once daily in the morning. The dose of liraglutide was determined using allometric inter-species scaling [17]. The treatment with liraglutide was started at 0.062 mg/kg/day (human equivalent dose of 0.6 mg/day) and increased weekly by 0.06 to the final dose of 0.186 mg/kg/day (human equivalent dose of 1.8 mg/day, submaximal dose for the treatment of obesity [21]), which was administered for 7 weeks. The dosing schedule, with weekly increases during the adaptation period, was designed to mirror Saxenda^®^ prescribing guidelines (https://www.saxenda.com/about-saxenda/dosing-schedule.html, accessed on 13 December 2023.), allowing gradual drug acclimatization and reducing gastrointestinal adverse effects.

Rats treated with liraglutide were monitored for potential adverse effects and exhibited only mild gastrointestinal side effects, primarily occasional diarrhea. No other visible adverse reactions (irritation of the skin or injection site, constipation, abdominal distension) or signs of distress or discomfort during injections were observed.

Rats in the exercise (E) and liraglutide plus exercise (L+E) groups performed a vertical ladder climbing training protocol as previously described [22,23]. The apparatus consisted of a 110 cm ladder with 2 cm grid steps inclined at 80°. Animals were first familiarized with the ladder by climbing without weights for two weeks. During the 7-week training period, sessions were conducted three times per week. Weights were attached to the base of the tail using adhesive tape, starting at 25% of body weight and progressively increasing to 50%. Each session consisted of repeated climbs from the bottom to the top, with a 60 s rest between trials, until the animal was unable to complete the climb after gentle tail stimulation, with each session limited to 10 min. Ladder climbing performance was estimated as the absolute number of successful climbs, as the measure of training volume, and the load volume (LV), as the measure of training intensity [24]. For each animal, LV was calculated as LV (g) = total successful climbs per session × weight load lifted (g). Both parameters were averaged over three separate sessions within a single week, with the difference calculated between the initial (25% b.w. load, post-acclimatization) and final week (50% b.w. load).

The study included only male rats because well-documented and expected major sex differences in physiological parameters (body, adipose and muscle mass), biochemical markers, and animals’ exercise capacity would substantially increase biological variability and reduce result interpretability within the limited sample size, in accordance with ARRIVE guidelines. Limiting to males thus enhanced statistical power and enabled a more controlled assessment of age-related changes.

### 2.4. Body Mass and Food Intake Measurements

Body mass was measured at the start and end of the experiment. Change in body mass was calculated as the difference between the final and initial values.

To enable accurate measurement of individual food intake and minimize stress, animals were individually housed for 48 h at the beginning of the experiment and again for 48 h, plus an additional 24 h adaptation period, a few days prior to sacrifice.

### 2.5. Sample Collection, Organ Weighing, and Serum Preparation

Twenty-four hours after the final treatment or exercise session, animals were fasted overnight and then euthanized by rapid guillotine decapitation, performed by trained institutional veterinary personnel. Because we measured sensitive metabolic, hormonal, and oxidative-stress endpoints, pharmacological anesthesia and analgesia were avoided to prevent documented pharmacologic confounding of these assays; inert-gas hypoxic methods were not used because hypoxia can alter oxidative-stress markers. Death was confirmed immediately after the procedure by the absence of cardiac activity and pupillary reflex. All procedures were approved by the Institutional Ethics Committee and the Veterinary Directorate of the Ministry of Agriculture, Forestry and Water Management of the Republic of Serbia (reference 000169446 2024 14841 002 001 000 001). The methods were justified in accordance with the 3R principles and applicable international guidelines, including the recommendations of FELASA (Federation of European Laboratory Animal Science Associations) and the AVMA (American Veterinary Medical Association). The liver, visceral intraperitoneal and bilateral retroperitoneal adipose depots and gastrocnemius muscles from both legs were excised and weighed (wet weight, g). Left and right retroperitoneal fat pads were combined, and gastrocnemius weights from both legs were averaged geometrically. Relative organ weights were expressed as g organ/g body weight.

Samples of distal pancreas and liver were collected for histology; aliquots of liver tissue were snap-frozen in liquid nitrogen and stored at −70 °C for further biochemical or molecular analyses.

Trunk blood was collected immediately after decapitation, allowed to clot at room temperature, and centrifuged at 3000× *g* for 15 min to obtain serum, which was stored at −70 °C.

### 2.6. Blood Analyses

Fasting serum samples were analyzed on an Abbott Alinity ci-series analyzer (Abbott Diagnostics, IL, USA). Glucose was measured by the hexokinase method; total cholesterol, triglycerides, and HDL-cholesterol were measured by enzymatic or direct methods. Non-HDL cholesterol was calculated as total minus HDL cholesterol. Serum insulin was determined using a commercial RIA kit (INEP, Belgrade, Serbia; sensitivity: 0.6 mIU/L; intra- and inter-assay CVs: 2.5% and 7.7%).

Insulin resistance indices were calculated as

HOMA-IR = [insulin (mIU/L) × glucose (mmol/L)]/22.5;

TyG = ln [triglycerides (mg/dL) × glucose (mg/dL)]/2.

An intraperitoneal glucose tolerance test (ipGTT) was performed 3–5 days before euthanasia following overnight fasting. Rats received glucose (2 g/kg i.p.), and blood glucose was measured at 0, 15, 30, 60, 90, and 120 min (GlucoSure AutoCode, Prizma, Kragujevac, Serbia). The glucose area under the curve (AUC) was calculated using GraphPad Prism 8 (GraphPad Software, San Diego, CA, USA).

Serum concentrations of biochemical parameters were measured using a BS-240 Vet Chemistry Analyzer (Shenzhen Mindray Animal Medical Technology Co., Ltd., Shenzhen Mindray Animal Medical Technology Co., Ltd., Shenzhen, China) with commercially available assay kits (BioSystems S.A., Barcelona, Spain). The following assays were performed: alanine aminotransferase (ALT) by the ALT/Glutamate Pyruvate Transaminase method; aspartate aminotransferase (AST) by the AST/GOT method; gamma-glutamyl transferase (GGT) by the gamma-glutamyl IFCC method; lactate dehydrogenase (LDH) by the lactate-to-pyruvate conversion method; albumin by the bromocresol green method; total bilirubin by the diazotized sulfanilic acid method; α-amylase by the direct substrate method; lipase by the DGGR method; alkaline phosphatase (ALP) by the 2-amino-2-methyl-1-propanol buffer IFCC method; calcium (Ca) by the calcium-arsenazo III method; and inorganic phosphorus (P) by the phosphomolybdate UV colorimetric method. Serum osteocalcin was determined on a cobas e411 immunoassay analyzer (Elecsys^®^ N-MID Osteocalcin, Roche Diagnostics, Mannheim, Germany).

### 2.7. Histological, Immunohistochemical, and Morphometric Analysis

Samples of the distal pancreas were fixed in neutral 10% formalin buffer for 24 h, while liver samples were fixed in Bouin’s fixative for 48 h. Fixed tissues were then dehydrated and embedded in Histowax^®^ (Histolab Product AB, Gothenburg, Sweden).

Hematoxylin–eosin (HE) staining was performed using standard protocols. Periodic acid–Schiff (PAS) staining assessed hepatic glycogen (0.5% periodic acid and Schiff reagent, 30 min each), while Picro–Sirius Red staining evaluated collagen deposition (Weigert’s hematoxylin, 8 min; Picro–Sirius Red, 1 h). Slides were dehydrated, cleared in xylene, and mounted with DPX. Following all histochemical procedures, tissue slides were dehydrated, cleared in xylene, and mounted with DPX. Digital images were obtained using a Leica DM4 B (Leica Microsystems CMS GmbH, Wetzlar, Germany) and Leica Application Suite X (LAS X) software, version 3.8.1.26810.

Morphometric analysis was performed on 3–5 randomly selected sections per animal (n = 6/group) using CAST stereological software, version 3.2.7.0 (Visiopharm; Denmark).

For histomorphometric analysis of the endocrine pancreas, the total islet area (µm^2^) was obtained by summing individual islet areas (all islets per section) and expressing this value as a percentage of the total pancreatic area, measured in HE-stained sections at 20× magnification using the mask tool in CAST software, version 3.2.7.0. PAS-stained livers were outlined at 4× magnification, and 50% of the tissue area was examined using systematic meander sampling. The volume density (V_V_, %) of PAS-positive cells was determined at 10× magnification with a 6 × 6 point grid. For Sirius Red-stained sections, collagen fibers were analyzed at 20× magnification using a 5 × 5 grid. Volume density was calculated as V_V_ (%) = (Pp/Pt) × 100, where Pp is the number of points hitting the target structure and Pt the total number of points.

For immunohistochemistry, previously published in detail [25], sections were incubated overnight at 4 °C with recombinant monoclonal rabbit anti-GLP1R antibody (EPR21819; 1:100; Abcam, Cambridge, UK). Immunodetection was performed using the VECTASTAIN^®^ ABC kit (rabbit IgG; Vector Laboratories, Newark, CA, USA) with the biotin–avidin system, following the manufacturer’s protocol. Sections were washed in 0.1 mol/L PBS (pH 7.2), counterstained with hematoxylin, and mounted in DPX medium (Sigma–Aldrich, Barcelona, Spain). DAB signal quantification in Langerhans islets was performed with ImageJ Fiji (v1.49j) using the IHC Profiler plugin. For each animal, six randomly selected islet images (2088 × 1550 pixels, 40× magnification) were analyzed. Optical density (OD) was calculated as OD = log (maximum intensity/mean intensity).

### 2.8. Hepatic Enzyme and Triglyceride Levels

Liver homogenates were prepared by mincing 200 mg of tissue and homogenizing in 10 volumes of sucrose–Tris–EDTA buffer (25 mmol/L sucrose, 10 mmol/L Tris–HCl, 0.1 mol/L EDTA; pH 7.4) at 4 °C using an Ultra–Turrax homogenizer (1500 rpm; 3 × 10 s). The homogenates were sonicated on ice (10 kHz; 3 × 10 s; Bandeline Sonopuls HD 2070) and centrifuged at 37,500 rpm for 90 min at 4 °C. Supernatants were collected for enzymatic assays. Antioxidant enzyme activities were measured in triplicate using a UV–1900i spectrophotometer (Shimadzu, Kyoto, Japan).

ALT and AST activities were determined in liver homogenates diluted to fit recommended assay range, using commercially available assay kits (Spinreact, S.A./S.A.U., Ctra. Santa Coloma, Spain), following the manufacturer’s instructions, and diluted to fit the recommended assay range. All measurements were performed in triplicate at 340 nm using a Shimadzu UV–1800 spectrophotometer (Shimadzu Corporation, Kyoto, Japan).

Triglycerides were measured in liver homogenates with a glycerol phosphate oxidase/peroxidase (GPO/POD) method, using the commercial assay kit (BioSystems S.A. of Barcelona, Spain,) on a BS-240 Vet Chemistry Analyzer (Shenzhen Mindray Animal Medical Technology Co., Ltd., Shenzhen, China).

### 2.9. Hepatic Gene Expression Analyses

Total RNA was isolated from small aliquots of liver (50 mg) by TRIzol reagent (Invitrogen, Carlsbad, CA, USA) according to the manufacturer’s instructions. The concentration and purity of the total RNA were assessed by a Nanophotometer^®^ N60 (Implen, Munich, Germany). Volume equivalent to 1 µg of RNA was used for reverse transcription to generate cDNA using a High-Capacity cDNA Reverse Transcription Kit (Applied Biosystems, Vilnius, Lithuania). Quantitative real-time PCR (qRT–PCR) was performed using cDNA samples in a real-time PCR machine ABI Prism 7000 (Applied Biosystems, Waltham, MA, USA) with SYBRGreen PCR master mix (Applied Biosystems, USA) for all examined gene expressions. Each sample was tested in duplicate. The expression levels of the target genes were calculated using the comparative 2−∆Ct method, using *Hprt* as housekeeping gene. The detailed protocol was described in [26]. The sequences of the used forward and reverse primers are listed in Supplementary Table S1.

### 2.10. Assessment of Oxidative Stress Parameters in the Liver

Oxidative stress parameters were analyzed as described previously [20] in liver homogenates that were prepared as described for hepatic enzyme activity. In brief, the activity of superoxide dismutase (SOD, EC 1.15.1.1) was measured by the epinephrine method [27] and expressed as U/mg protein. Catalase (CAT, EC 1.11.1.6) activity was assessed by the decomposition rate of hydrogen peroxide [28] and expressed as µmol H_2_O_2_/min/mg protein. Glutathione peroxidase (GSH–Px, EC 1.11.1.9) activity was determined by NADPH oxidation in the presence of t-butyl hydroperoxide [29] and expressed as nmol NADPH/min/mg protein. Glutathione reductase (GR, EC 1.8.1.7) activity was measured via NADPH-dependent reduction of oxidized glutathione (GSSG) [30] and expressed as nmol NADPH/min/mg protein. Glutathione S-transferase (GST, EC 2.5.1.18) activity was evaluated using the method of [30] and expressed as nmol GSH/min/mg protein.

Non-enzymatic parameters included total glutathione (GSH) content (µmol/g wet mass) and sulfhydryl (SH) group concentration, which was estimated using the method of [26] and expressed as nmol/g wet mass. Lipid peroxidation was estimated by quantifying thiobarbituric acid reactive substances (TBARS) according to [31] and expressed as nmol TBARS/mg wet mass.

Total oxidant status (TOS) and total antioxidant status (TAS) were measured using colorimetric commercial kits (Elabscience, Houston, TX, USA) following the manufacturer’s instructions. TOS was expressed as μmol H_2_O_2_ equivalents/g wet mass, while TAS was expressed as mmol Trolox equivalents/g wet mass. The oxidative stress index (OSI) was calculated as (TOS/TAS) × 100 and expressed in arbitrary units.

### 2.11. Hepatic Measurements of Oxysterols and Cholesterol

Aliquots of liver tissue were dried in a Speedvac concentrator (Savant DNA120, Thermo Scientific GmbH, Karlsruhe, Germany) at room temperature and the dry weight was taken as the basis for calculation of the cholesterol and oxysterol concentrations. After chloroform/methanol extraction (2:1) of cholesterol and oxysterols, an aliquot of 100 µL of the extract was used for further separation of the (di-)trimethylsilylated sterol ethers on a DB–XLB 30 m × 0.25 mm i.d. × 0.25 µm film thickness (J&W Scientific Alltech, Folsom, CA, USA).

Gas chromatographic separation and detection of cholesterol and 5α-cholestane (ISTD) was performed in a Hewlett–Packard (HP) 6890 Series GC-system (Agilent Technologies, Palo Alto, CA, USA) equipped with a flame ionization detector (FID).

The oxysterols (2Hx–oxysterols, ISTD) were detected by highly specific and sensitive mass spectrometry in the selected ion monitoring mode (MS–SIM) in an HP 6890N Network GC system (Agilent Technologies, Waldbronn, Germany) connected to a direct capillary inlet system to a quadrupole mass-selective detector HP5975B inert MSD (Agilent Technologies, Waldbronn, Germany). The identity of all sterols was proven by comparison with the full-scan mass spectra of authentic compounds. A detailed description of the analytical protocol was previously reported [26].

### 2.12. Statistical Analysis

Data were analyzed using the GraphPad Prism Software (v.8, San Diego, CA, USA). The normality of the distribution was assessed with the Shapiro–Wilk and Kolmogorov–Smirnov tests. A one-way ANOVA followed by Tukey’s post hoc test was used for comparisons between the experimental groups. Body weight, food intake and number of successful climb differences between the start and end of the experiment were analyzed by two-way repeated-measures ANOVA followed by Sidak’s test. Total load volume changes between two experimental time points (25% vs. 50% b.w. load weeks) were analyzed using the non-parametric Wilcoxon matched-pair signed-rank test. Endpoint comparisons between exercise (E) and liraglutide + exercise (L+E) groups used the Mann–Whitney U test. Statistical significance was set at * *p* < 0.05, ** *p* < 0.01, *** *p* < 0.001, and **** *p* < 0.0001. Results are expressed as the mean ± standard error of the mean (SEM).

## 3. Results

### 3.1. Physiological and Biochemical Parameters

**Figure 2:**
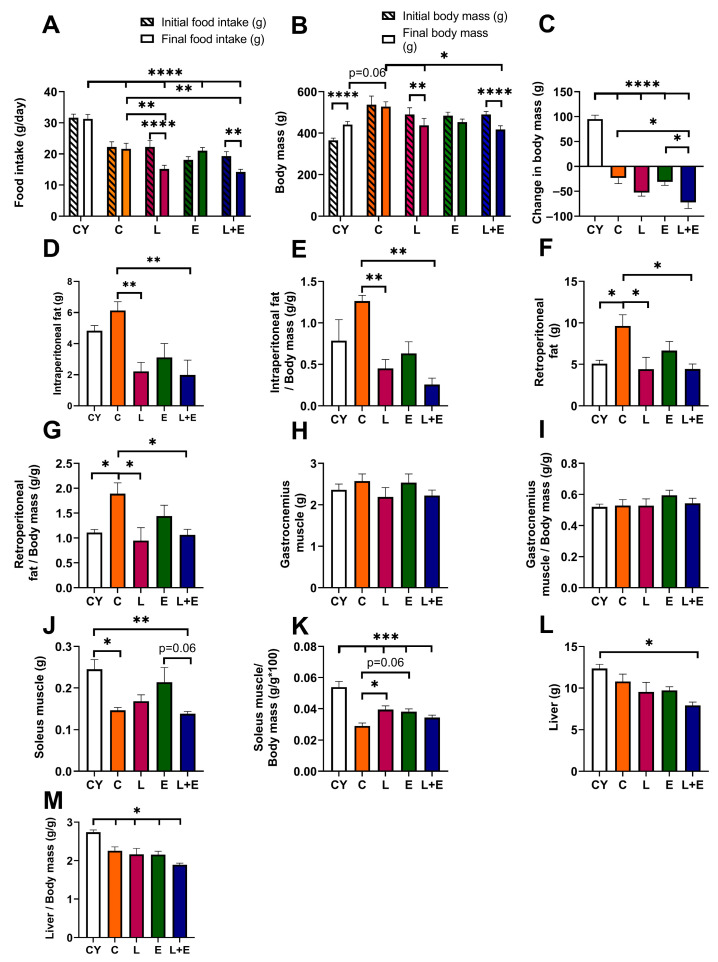
Food intake (**A**), body mass (**B**) and change in body mass (**C**) at the beginning and end of the experiment; absolute (**D**) and relative (**E**) intraperitoneal adipose tissue weight; absolute (**F**) and relative (**G**) retroperitoneal adipose tissue weight; absolute (**H**) and relative (**I**) gastrocnemius muscle weight; absolute (**J**) and relative (**K**) soleus muscle weight; and absolute (**L**) and relative (**M**) liver weight in young adult (CY) and middle-aged (C) control, liraglutide-treated (L), exercise (E), and combined liraglutide plus exercise (L+E) groups of male rats. Data are presented as mean ± SEM (n = 8/group). A two-way repeated-measures ANOVA was used to analyze body mass and food intake at start and end time points, followed by a post hoc Sidak test for pairwise comparisons, while between-group comparisons were analyzed using one-way ANOVA followed by Tukey’s test. Statistical significance is indicated as * *p* < 0.05, ** *p* < 0.01, *** *p* < 0.001, **** *p* < 0.0001.

Analysis of food intake at the start and end of the experiment for each group revealed significant reductions in the L (*p* < 0.001) and L+E (*p* < 0.01) groups (Figure 2A). Further between-group comparisons showed age-related differences, with all middle-aged groups consuming less than CY (*p* < 0.0001), with L and L+E consuming less than C (*p* < 0.01) (Figure 2A).

The CY group showed a significant increase in body mass (*p* < 0.0001) from the start to the end of the experiment; the C and E groups remained stable, while the L (*p* < 0.01) and L+E (*p* < 0.0001) groups lost weight (Figure 2B). Statistical analysis of weight change (Figure 2C) confirmed that all middle-aged groups lost weight compared to CY (*p* < 0.0001), while the L+E group lost more weight than both the C (*p* < 0.05) and E (*p* < 0.05) groups.

When analyzing absolute and relative visceral adipose tissue weights, the C group exhibited increased retroperitoneal fat compared to the CY group (*p* < 0.05), while the L and L+E groups showed reduced intraperitoneal (*p* < 0.01) and retroperitoneal fat weights (*p* < 0.05) compared to the C group (Figure 2D–G). Gastrocnemius muscle weight remained unchanged across all groups (Figure 2H,I). However, absolute soleus muscle weight was lower in C and L+E groups compared to CY (*p* < 0.05, Figure 2J), reaching a trend toward a decrease when comparing L+E and E groups (*p* = 0.06). Relative soleus muscle weight was lower in all middle-aged groups compared to CY (*p* < 0.001), being increased (*p* < 0.05) in L and E (*p* = 0.06) compared to C (Figure 2K). Absolute liver weight was significantly lower in the L+E group compared to CY (*p* < 0.05; Figure 2L), whereas relative liver weights were decreased in all experimental groups compared to CY (*p* < 0.05; Figure 2M).

In the E group (n = 8), animals completed an average of 3.1 ± 0.2 successful climbs per session at 25% body weight, resulting in an initial total load volume (LV) of 296.5 ± 29.7 g. By the final week, with a 50% body weight load, the number of successful climbs increased to 5.6 ± 0.8 per session, yielding an LV of 1286 ± 232 g, representing a 4.38-fold increase (*p* < 0.05). In the L+E group (n = 8), animals completed 3.5 ± 0.4 climbs per session at 25% body weight, corresponding to an initial LV of 290.8 ± 41.9 g. By the final week at 50% load, successful climbs increased to 4.9 ± 0.7 per session, resulting in an LV of 929.1 ± 131 g, a 3.2-fold increase (*p* < 0.05). No significant declines were observed between the final LVs of the E and L+E groups.

Middle-aged controls had higher total cholesterol and non-HDL cholesterol levels compared to young adult controls (*p* < 0.05 and *p* < 0.01, respectively), while serum triglyceride levels were at the upper range of CY (Table 1). Neither liraglutide, exercise, nor their combination significantly altered cholesterol or lipoprotein levels in aged animals. However, serum triglyceride levels were decreased in the L+E group compared to the C (*p* < 0.05) and E (*p* < 0.05) groups (Table 1).

Fasting serum glucose, insulin and HOMA–IR did not differ significantly among groups. Glucose tolerance, assessed by the intraperitoneal glucose tolerance test, is illustrated by the glucose concentration curves shown in Supplementary Figure S2. The curve shape and calculated AUC did not differ significantly between experimental groups (Table 1). However, the TyG index was significantly improved in the L+E group compared to the C (*p* < 0.01) and E (*p* < 0.05) groups (Table 1).

Among the hepatogram parameters analyzed (ALT, AST, GGT, LDH, serum albumin, and bilirubin), only serum albumin was significantly lower (*p* < 0.05) in both the C and L groups compared to the young adult controls. Notably, serum albumin levels in the L group were comparable to those in the age-matched middle-aged controls (Table 1). Additionally, serum lipase and amylase levels, markers of exocrine pancreas function, did not differ significantly between groups (Table 1).

Serum concentrations of osteocalcin, markers of bone formation and osteoblast activity, alkaline phosphatase activity, and calcium and phosphorus concentrations were measured to examine possible effects of liraglutide (and expected weight loss) on parameters of bone formation and turnover (Table 1). Age-related decreases in osteocalcin and *p* levels were demonstrated in all middle-aged groups compared to CY (*p* < 0.05), without significant changes in other experimental groups.

### 3.2. Histomorphometric Analysis of Endocrine Pancreas and Immunohistochemical Expression of GLP–1R

**Figure 3:**
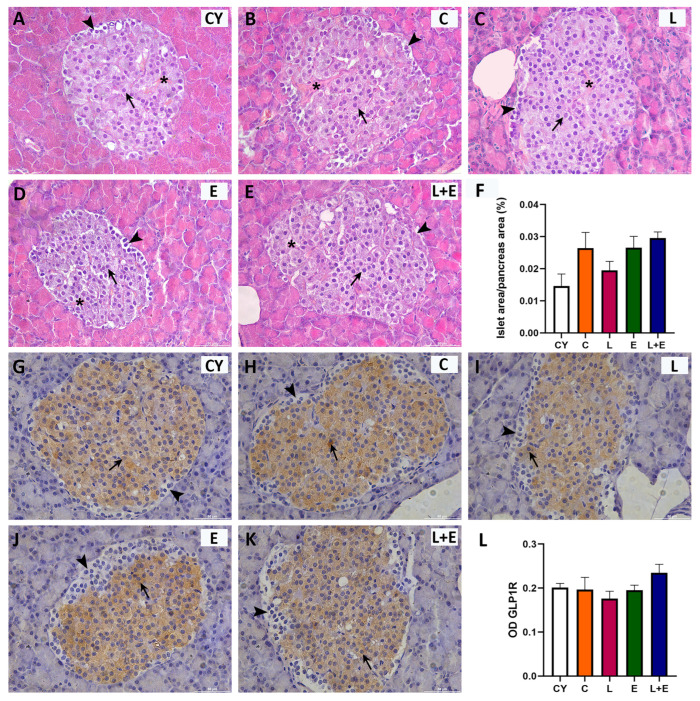
Representative micrographs of Islets of Langerhans stained with hematoxylin and eosin (HE; (**A**–**E**)) or immunohistochemically for glucagon-like peptide 1 receptor (GLP–1R; (**G**–**K**)) from young adult (CY) and middle-aged (C) control, liraglutide-treated (L), exercise (E), and combined liraglutide plus exercise (L+E) groups of male rats. Scale bar: 100 mm. Morphometric analysis of Langerhans islet area per pancreas area (µm^2^; (**F**)) and optical density (OD; (**L**)) of GLP1–R immunostaining in the Langerhans islets (**B**); morphometric data are presented as mean ± SEM (n = 6/group) and were analyzed by one-way ANOVA followed by Tukey’s test. Arrows indicate beta cells, arrowheads indicate alpha cells, and the asterisks mark blood vessels.

Histopathological examination of the Islets of Langerhans revealed regular shape and normal vascularization across all groups (Figure 3A–E). The islets showed typical architecture, with a core of large β cells (Figure 3A–E, arrows) and α cells localized to the periphery, smaller and polygonal in shape (Figure 3A–E, arrowhead). Morphometric analysis indicated preserved islet mass (Figure 3F). Immunohistomorphometry showed strong granular GLP-1R immunopositivity in the cytoplasm of β cells (Figure 3G–K, arrows), while peripheral α cells were negative (Figure 3). This pattern and GLP-1R expression levels were consistent across groups (Figure 3L). Furthermore, histological examination of the exocrine pancreatic acini revealed preserved structure in all experimental groups, with no evidence of ductal destruction, inflammatory infiltration, or fibrosis.

### 3.3. Histomorphometric Analysis of Liver, Hepatic Enzyme Activities, and Oxidative Stress Parameters

**Figure 4:**
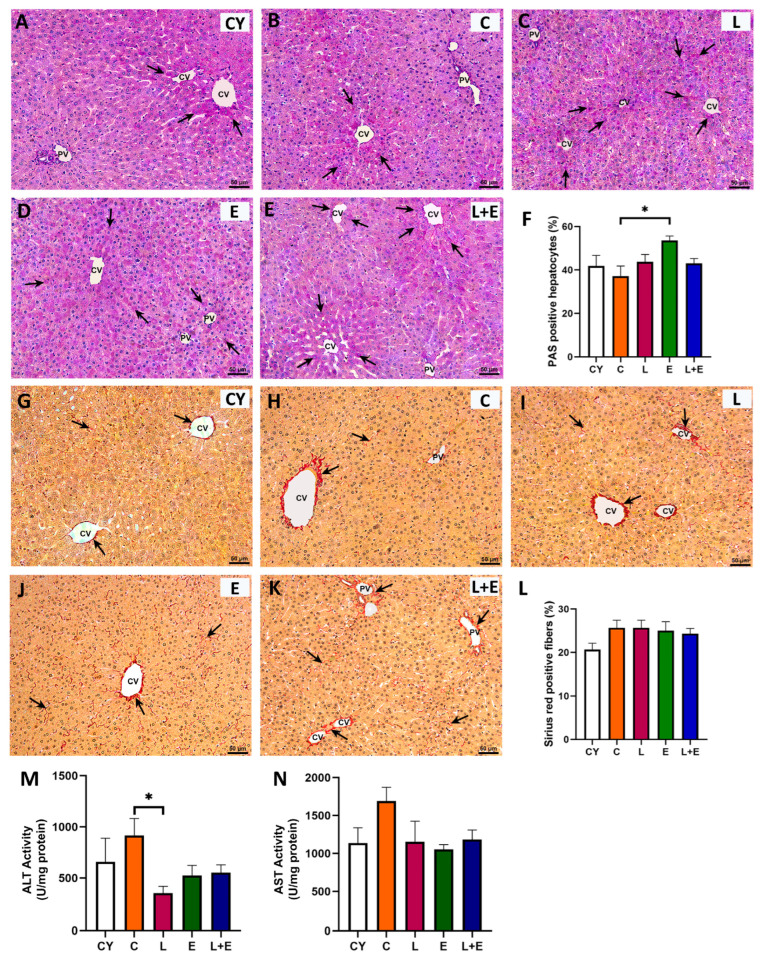
Representative micrographs of the liver tissue (PV = portal vein; CV = central vein) stained with periodic acid–Schiff (PAS; (**A**–**E**); arrows point to magenta stained hepatocytes) and percentage of PAS-positive hepatocytes (**F**). Representative micrographs of the liver tissue stained with Sirius red (**G**–**K**) and percentage of Sirius red-positive fibers (**L**), hepatic levels of alanine (ALT; (**M**)) and aspartate (AST; (**N**)) aminotransferase from young adult (CY) and middle-aged (C) control, liraglutide-treated (L), exercise (E), and combined liraglutide plus exercise (L+E) groups of male rats. Data are presented as mean ± SEM (n = 8/group). Statistical significance was determined by one-way ANOVA followed by Tukey’s test; * *p* < 0.05.

Histological examination of HE-stained liver sections showed normal lobular architecture with well-defined portal triads and radial hepatocyte arrangement around central veins, without histopathological alterations or GLP-1 immunopositivity (Supplementary Figure S3). PAS staining revealed zonal glycogen distribution consistent with fasting, highest near central veins in most groups (Figure 4A–E, arrows). However, the E group displayed more uniform PAS positivity along the periportal–pericentral axis, with morphometry confirming a significant increase in PAS-positive hepatocytes compared to controls (Figure 4F, *p* < 0.05). Sirius Red staining showed predominantly perivascular and lesser perisinusoidal collagen fibers (Figure 4G–K), with slightly increased perivascular collagen in middle-aged livers but no fibrosis. Quantification showed no significant differences in collagen deposition between groups (Figure 4L).

Statistical analysis of hepatic ALT and AST enzyme activities (Figure 4M,N) revealed an age-related increase in ALT levels (*p* < 0.05) when comparing the young adult controls (CY) to the middle-aged group (C). Additionally, a significant decrease in ALT activity (*p* < 0.05) was observed in the L group compared to the C group (Figure 4M).

Hepatic oxidative stress parameters are summarized in Table 2. Compared to young adults, middle-aged controls showed reduced GSH-Px (*p* < 0.05) and GST (*p* < 0.05) activities, decreased total GSH (*p* < 0.05), and increased SH groups (*p* < 0.05) and TOS (*p* < 0.05), along with elevated TAS (*p* < 0.05), indicating compensatory antioxidant responses. Liraglutide treatment increased GST activity (*p* < 0.05 vs. C), decreased GSH (*p* < 0.05 vs. both C and CY) and LPO (*p* < 0.05 vs. both C and CY), increased TOS (*p* < 0.05 vs. CY; the same parameter decreased *p* < 0.05 vs. E), elevated TAS (*p* < 0.05 vs. CY), and reduced OSI (*p* < 0.05 vs. E). The exercise group (E) showed reduced GSH-Px (*p* < 0.05 vs. CY), decreased LPO (*p* < 0.05 vs. C and CY), elevated TOS (*p* < 0.05 vs. C and CY), increased TAS (*p* < 0.05 vs. CY), and unchanged OSI. Compared to E group, L+E group had higher CAT and GR activities (*p* < 0.05 vs. E), lower LPO (*p* < 0.05 vs. E), and reduced TOS and OSI (*p* < 0.05 vs. E). The combined treatment (L+E) showed the greatest CAT (*p* < 0.05 vs. C, CY, E), GR (*p* < 0.05 vs. C, E), and GST (*p* < 0.05 vs. C) activities, decreased LPO (*p* < 0.05 vs. C, CY), elevated TAS (*p* < 0.05 vs. CY), and reduced OSI (*p* < 0.05 vs. E), confirming liraglutide’s protective effect against exercise-induced oxidative imbalance.

### 3.4. Gene Expressions in the Liver

**Figure 5:**
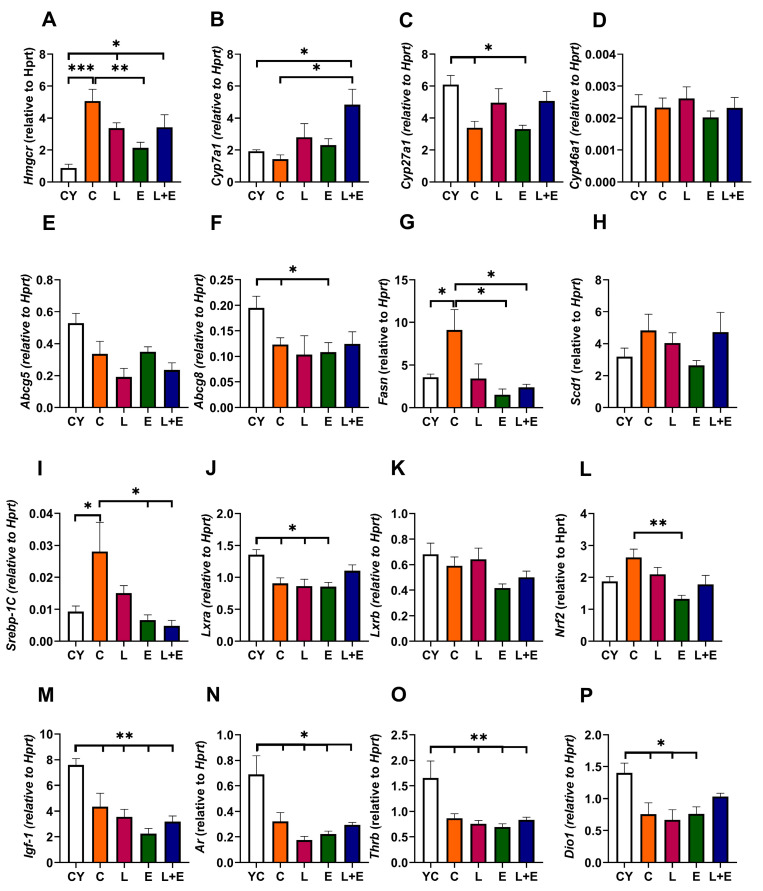
mRNA expression of *Hmgcr* (**A**), *Cyp7a1* (**B**), *Cyp27a1* (**C**), *Cyp46a1* (**D**), *Abcg5* (**E**), *Abcg8* (**F**), *Fasn* (**G**), *Scd1* (**H**), Srebp–1c (**I**), *Lxr*a (**J**), *Lxr*b (**K**), *Nrf2* (**L**), *Igf-1* (**M**), *Ar* (**N**), *Thrb* (**O**) and *Dio1* (**P**) in the liver of young adult (CY) and middle-aged (C) control, liraglutide-treated (L), exercise (E), and combined liraglutide plus exercise (L+E) groups of male rats; Data are presented as mean ± SEM, n = 8/group. Statistical significance was determined by one-way ANOVA followed by Tukey’s test; * *p* < 0.05, ** *p* < 0.01, *** *p* < 0.001.

Aging significantly upregulated hepatic *Hmgcr* expression, which encodes the rate-limiting enzyme in cholesterol synthesis, in all middle-aged groups except E (CY vs. C, *p* < 0.001; CY vs. L and L+E, *p* < 0.05; E vs. C, *p* < 0.01) (Figure 5A). *Cyp7a1*, which encodes the rate-limiting enzyme in the main pathway of cholesterol degradation to bile acids and is an *LXR* target, was unchanged with age but upregulated in L+E vs. C and CY (*p* < 0.05) (Figure 5B). *Cyp27a1,* which encodes the enzyme that initiates an alternative pathway of cholesterol degradation to bile acids, was downregulated in C and E groups compared to CY (*p* < 0.05) (Figure 5C), while *Cyp46a1* transcript levels remained unaltered (Figure 5D). Furthermore, gene expressions of *Abcg5* and *Abcg8*, which encode cholesterol transporters that mediate cholesterol efflux into bile acids and are targets of LXR, were assessed (Figure 5E,F); only *Abcg8* expression was significantly downregulated in the C, L, and E groups compared to the CY group (*p* < 0.05) (Figure 5F). *Fasn*, key in lipogenesis and a *Srebp-1c* target, showed an age-related increase (CY vs. C, *p* < 0.05), but was lower in exercised groups (E, L+E) compared to C (*p* < 0.05) (Figure 5G). *Scd1* expression was unaltered (Figure 5H). *Srebp-1c* mirrored *Fasn*, upregulated with age (C vs. CY, *p* < 0.05) and reduced in E, L+E vs. C (*p* < 0.05) (Figure 5I). *Lxr*a was downregulated with age except in L+E (*p* < 0.05), while *Lxr*b was unchanged (Figure 5J,K). *Nrf2*, the primary transcription factor of antioxidant defense [28], was downregulated in E vs. C (*p* < 0.05) (Figure 5L).

To examine age-related changes and hormone responsiveness in the liver, we examined expressions of growth hormone-regulated *Igf-1*, androgen receptor *Ar*, and thyroid hormone-regulated *Trb,* which were all significantly lower in middle-aged groups vs. CY (*p* < 0.01), while *Dio1* was decreased in all middle-aged groups except L+E (*p* < 0.05) (Figure 5M–P).

### 3.5. Oxysterols, Cholesterol and Triglyceride Levels in the Liver

**Figure 6:**
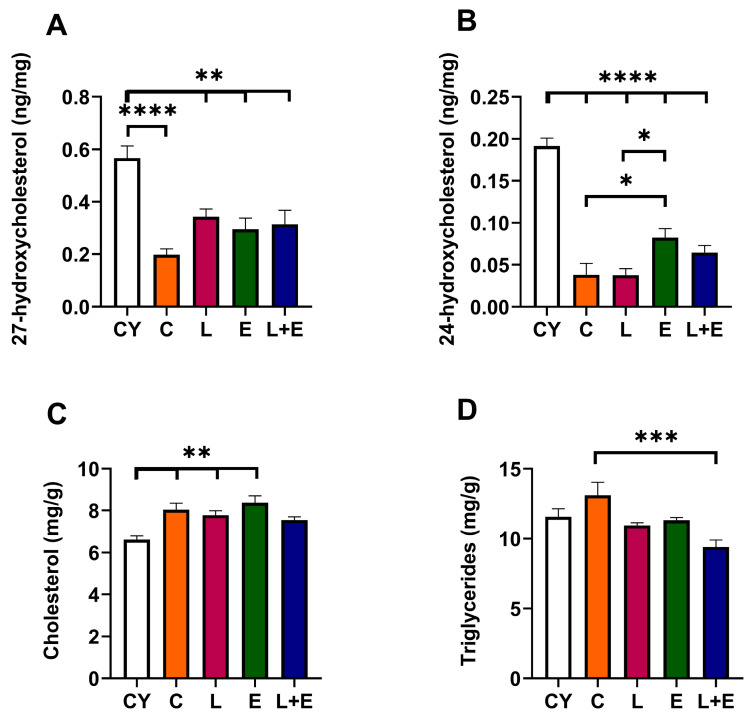
Concentrations of 27-hydroxycholesterol (**A**), 24-hydroxycholesterol (**B**), cholesterol (**C**) and triglycerides (**D**) in the liver of young adult (CY) and middle-aged control (C), liraglutide-treated (L), exercise (E), and combined liraglutide plus exercise (L+E) male rats. Data are presented as mean ± SEM, n = 8/group. Statistical significance was determined by one-way ANOVA followed by Tukey’s test; * *p* < 0.05, ** *p* < 0.01, *** *p* < 0.0001, **** *p* < 0.0001.

Finally, we analyzed hepatic oxysterol levels, the enzymatic byproducts of cholesterol metabolism, 24- and 27-hydroxycholesterol. The oxysterols act as the primary endogenous ligands for liver X receptors (LXRs), promoting transcription of key genes essential for maintaining cholesterol homeostasis. 27-hydroxycholesterol levels were lower in all middle-aged groups compared to the CY group (CY vs. C, *p* < 0.0001; CY vs. L, E or L+E, *p* < 0.01). Notably, exercise alone led to an increase in 24-hydroxycholesterol levels (*p* < 0.05 vs. C and L). Moreover, we examined hepatic cholesterol levels, which were higher in all middle-aged groups compared to CY (*p* < 0.01; Figure 6C) except in the L+E group, where they remained unchanged. Hepatic triglyceride levels were decreased in L+E group compared to C (*p* < 0.001).

## 4. Discussion

This study aimed to investigate the effects of liraglutide and strength–endurance exercise training on metabolic and liver health, particularly focusing on hepatic oxidative stress, lipid metabolism, and insulin sensitivity and liver function markers in a middle-aged rat model.

At the start of the experiment, middle-aged male rats fed ad libitum exhibited early age-related metabolic decline, including 40% higher body weight than young controls—a difference that narrowed to 15% over time, consistent with mild spontaneous obesity [32,33]—alongside reduced food intake, reflecting age-associated changes in appetite regulation and metabolic efficiency [34]. They also showed increased visceral fat, reduced soleus muscle mass, elevated total and non-HDL cholesterol, and preserved serum glycemic parameters. These findings align with previous reports linking aging to hypercholesterolemia and mild insulin resistance in rodents [25,35,36].

Liraglutide at a submaximal dose, with or without exercise training, reduced food intake, body weight, and visceral fat while preserving gastrocnemius and soleus muscle mass, indicating favorable tissue-specific remodeling in middle-aged male rats. The obtained reduced food intake and associated weight and visceral adipose tissue loss are consistent with its role as a GLP-1 receptor agonist that enhances leptin sensitivity and promotes anorexigenic neuropeptides such as POMC and CART in the hypothalamus [21,37]. Our findings suggest that submaximal liraglutide dosing may balance efficacy with safety, limiting muscle loss while improving metabolic outcomes in aging [12,38].

In this study, the ladder-climbing exercise training [22,23] was adapted for middle-aged animals, incorporating progressive resistance training with repeated climbs and brief rests until voluntary fatigue, thus modeling strength training with endurance elements. This regimen induced modest hypertrophy in the soleus muscle, while gastrocnemius mass remained unchanged, consistent with reports that such training predominantly engages muscles performing concentric contractions with minimal eccentric loading [22]. Given its postural role and high proportion of slow-twitch fibers, the soleus is particularly responsive to both age-related neuromuscular and nutritional decline and training stimuli [39]. Ladder-climbing performance, assessed by the number of successful climbs and total load volume, confirmed training-related improvements in both the E and L+E groups, although gains were slightly (but not significantly) attenuated in the L+E group. Animal studies using GLP-1 receptor agonists have demonstrated confounding effects on skeletal muscle—some reporting preservation of fiber integrity, improvements in oxidative capacity, and enhanced endurance performance, while others describe neutral or adverse outcomes—depending on factors such as age, drug dose, metabolic status, and nutritional conditions (reviewed in [40]). The current analyses of muscle mass and exercise performance provide limited insight into liraglutide’s effects on skeletal muscle, as they rely on gross morphological outcomes without accompanying histological or molecular assessments. Future studies should combine fiber type and adipose/vascular morphometry and comprehensive strength and behavioral tests to clarify how liraglutide, with or without exercise, affects skeletal muscle structure and function in our model.

Histological findings in both the exocrine and endocrine pancreas showed no evidence of pancreatic injury or pancreatitis. This aligns with previous studies demonstrating the pancreatic safety profile of liraglutide [41]. Further histomorphometric analysis of pancreatic Langerhans islets showed preserved architecture and islet mass in all middle-aged groups relative to young controls, which is critical for proper hormone secretion [38]. Cytoplasmic GLP–1R immunopositivity was clearly evident in the cells displaying the morphological and nuclear characteristics of β cells, whereas expression in α cells was below IHC detection limits, consistent with previous immunodetection studies [42,43]. Serum parameters of glucose regulation and insulin resistance, as well as lipase and amylase levels, remained within normal ranges, additionally supporting that liraglutide was well tolerated in non-diabetic animals. These results are consistent with evidence that GLP-1 receptor agonists enhance insulin secretion only under hyperglycemic conditions [44].

The liver was a key focus of this study. In middle-aged rats, liver histology appeared largely preserved, without any sign of fatty liver or overt hepatic pathology. However, decreased hepatic GSH and glutathione-related enzyme activities, in line with literature data, may reflect a decreased liver capacity to counteract oxidative stress [45,46]. Age-associated GSH depletion lowers cellular thiol availability and GSH-Px activity, weakening antioxidant defense, while compensatory increases in protein-bound sulfhydryl groups may occur [47]. The observed changes, together with reduced expression of hormone-responsive genes and elevated hepatic ALT levels, may reflect increased vulnerability of the middle-aged liver and heightened risk of oxidative stress and metabolic dysregulation [2], already manifested at this early stage as increased hepatic and serum cholesterol and non-HDL.

GLP-1 receptor immunopositivity was undetectable in hepatic tissue, consistent with reports questioning its expression level and distribution [5,6]. This likely reflects low or cell-specific GLP-1R expression below IHC detection limits, suggesting that GLP-1′s hepatic effects are primarily indirect.

Exercise training alone resulted in increased hepatic PAS positivity and elevated oxidative stress markers, indicating physiological adaptations to high-intensity training [48]. Elevated hepatic glycogen has been reported to enhance exercise capacity [49] and restrict the conversion of glucose to lipids, thereby contributing to improved insulin sensitivity following high-intensity training [50]. The lipogenic genes Srebp1-c and *Fasn* were downregulated in the E group compared to the C group, despite no significant changes in hepatic and serum triglyceride levels. Conversely, a similar training regimen has been shown to induce weight loss and lipid-lowering effects in diet-induced obese rats [19], suggesting that baseline adiposity may influence the metabolic outcomes of exercise interventions. The observed hepatic oxidative stress in the E group could be interpreted as a hormetic response, in which mild pro-oxidant stimulation activates protective and repair pathways [51]. However, keeping in mind the downregulation of *Nrf2* gene expression, the primary transcription factor of antioxidant defense [52], these changes probably reflected a too-intense training modality for middle-aged rats [53].

Liraglutide alone reduced hepatic ALT and visceral fat, mainly improving oxidative stress parameters compared to E group. The antioxidative effects were most prominent in the L+E group, where catalase and glutathione reductase activities increased, and lipid peroxidation and TOS decreased beyond the baseline levels of middle-aged controls. This highlights liraglutide’s ability to mitigate exercise-induced oxidative stress in the middle-aged liver, consistent with its reported antioxidant and hepatoprotective actions in animal models of liver injury and metabolic disease [54,55].

The L+E group showed reduced hepatic and serum triglyceride levels and induced visceral fat loss. Transcript levels of lipogenic genes (*Srebp-1c* and *Fasn*) were downregulated in both E and L+E groups, suggesting a synergistic effect where liraglutide-mediated visceral fat reduction limited lipid substrates [56], contributing to reduced hepatic load and triglyceride synthesis and secretion. Although L+E lowered triglycerides, it only modestly affected hepatic cholesterol and did not reverse serum cholesterol increases, indicating distinct regulation mechanisms.

Aging increased hepatic and serum cholesterol and non-HDL, coinciding with higher hepatic *Hmgcr* expression, lower *Cyp27a1* expression, reduced levels of 27- and 24-hydroxycholesterols, and downregulated *Lxra* and *Abcg8* expression. Taken together, these results clearly indicate age-related dysregulation of cholesterol metabolism, specifically in the liver. Such changes involve complex alterations in cholesterol metabolism, including increased synthesis, reduced degradation, and impaired transport, in both rodent models and humans [57].

Strength–endurance training partially reversed hepatic 24-hydroxycholesterol, without affecting hepatic *Cyp46a1* expression. This group also showed reduced *Hmgcr* transcript levels compared to middle-aged controls, while *Cyp27a1*, *Lxra*, and *Abcg8* remained low, reflecting age-related patterns, resulting in unchanged hepatic and serum cholesterol levels. Although the brain is the primary site of 24-hydroxycholesterol production via CNS-specific CYP46A1, the liver primarily eliminates it, with ~30% of this oxysterol synthesized in the rat liver [58]. The aerobic exercise increased levels of CYP46A1 as well as its metabolite 24-hydroxycholesterol in an APP/PS1 mouse model of Alzheimer’s disease [59].

Combined L+E provided some positive transcriptional changes: it improved *Cyp7a1* beyond the baseline, and partly reversed *Hmgcr*, *Lxra* and *Abc8* gene expressions compared to young adults, leading to modestly improved hepatic cholesterol without major serum cholesterol changes. Cholesterol homeostasis regulation is complex, involving multiple pathways, with LXR-mediated signaling playing a central role. LXRα is the main liver isoform, and impaired LXRα signaling is associated with hepatic cholesterol accumulation, liver dysfunction, and progression of metabolic-associated steatohepatitis [60,61]. The age-related decline in 27- and 24-hydroxycholesterols, the principal endogenous ligands and activators of LXR-mediated signaling [62], may contribute to the observed changes. Furthermore, multi-hormonal decline with aging in our model [26,63], particularly thyroid hormone deficiency, may contribute to cholesterol dysregulation [64] and downregulation of *Lxra* [65]. Notably, thyroid hormone-dependent *Dio1* expression was modestly improved in the L+E group. The role of LXR signaling in aging and associated pathologies is tissue-specific and complex [66], and further studies are needed to clarify its relevance to hepatic aging.

LXRα, aside from mediating cholesterol homeostasis, contributes to insulin-mediated lipogenesis via *Srebp-1c* activation, with fatty acid synthesis genes showing greater sensitivity to *LXR*α regulation than cholesterol metabolism [61,67]. Moreover, *Srebp-1c* expression can be modulated independently of *LXR*α through other mechanisms, including reduced free fatty acid flux or oxidative stress [55,68], contributing to lower hepatic and serum triglycerides in L+E. Further studies using higher doses of liraglutide, additional cholesterol-lowering agents, or supplements are needed to better explore cholesterol reduction strategies in this model of early metabolic dysfunction.

## 5. Conclusions

This study shows that liraglutide combined with strength–endurance exercise synergistically improves hepatic oxidative stress and lipid metabolism in middle-aged male rats, without apparent adverse effects. Liraglutide reduced weight gain and visceral fat and preserved muscle mass and ladder-climbing performance, while exercise enhanced glycogen storage and suppressed insulin-regulated lipogenic genes. Together, these interventions enhanced antioxidant defenses and reduced lipogenic gene expression and lipid peroxidation, favoring triglyceride reduction over cholesterol. Age-related cholesterol rises, linked to elevated hepatic oxysterols, reduced transcript levels of *Lxra/Abcg8*, upregulated *Hmgcr*, and downregulated thyroid responsiveness, were partly reversed by L+E, modestly lowering hepatic but not serum cholesterol. Future studies incorporating female rats in the same experimental setup are needed to delineate sex-specific responses to liraglutide, with and without exercise training. While our preclinical findings highlight promising biological reserve recruitment and mechanistic improvements, the justification for the early clinical use of liraglutide requires rigorous human trials to evaluate its benefits and risks, ensuring appropriate application in the target population.

## Figures and Tables

**Table 1:** Serum biochemical parameters in young adult (CY) and middle-aged (C) control, liraglutide-treated (L), exercise (E), and combined liraglutide plus exercise (L+E) groups of male rats.

	CY	C	L	E	L+E
Cholesterol (mg/dL)	56.0 ± 3.8 ^C^	86.9 ± 8.6 ^CY^	89.3 ± 4.9	77.5 ± 5.9	77.6 ± 4.2
HDL (mg/dL)	18.2 ± 1.6	19.8 ± 1.6	19.2 ± 1.7	19.8 ± 1.3	19.8 ± 1.6
Non–HDL (mg/dL)	37.8 ± 2.2 ^C^	67.2 ± 8.4 ^CY^	70.4 ± 4.3	62.6 ± 3.5	57.9 ± 3.1
Triglycerides (mg/dL)	88.4 ± 12.5	103.4 ± 11.7 ^L+E^	69.2 ± 7.3	89.5 ± 8.0 ^L+E^	55.6 ± 3.7 ^C,E^
Glucose (mg/dL)	86.1 ± 3.5	84.6 ± 2.3	83.8 ± 4.5	89.0 ± 4.3	85.2 ± 3.8
ipGTT AUC	1055 ± 70	1304 ± 144	1077 ± 116	1156 ± 77	1027 ± 105
Insulin (mlU/L)	21.7 ± 1.6	22.20 ± 4.9	16.4 ± 1.7	22.1 ± 1.6	21.5 ± 2.6
HOMA–IR	4.9 ± 0.3	5.0 ± 1.2	3.4 ± 0.3	4.1 ± 0.7	4.8 ± 0.9
TyG index	4.4 ± 0.1	4.5 ± 0.1 ^L+E^	4.3 ± 0.1	4.5 ± 0.1 ^L+E^	4.2 ± 0.0 ^C,E^
ALT (U/L)	45.5 ± 6.6	69.89 ± 4.4	64.2 ± 9.9	79.0 ± 9.4	60.4 ± 3.7
AST (U/L)	273.0 ± 40.7	292.1 ± 36.6	273.0 ± 32.2	276.6 ± 27.04	242.4 ± 19.9
GGT (U/L)	5.7 ± 0.7	13.4 ± 3.5	14.1 ± 3.4	11.4 ± 2.1	10.0 ± 2.0
LDH (U/L)	4006.0 ± 92.0	3763.0 ± 232.6	3127.0 ± 251.7	3880.0 ± 301.1	3561.0 ± 319.2
Albumin (g/L)	42.9 ± 1.0	35.2 ± 2.0 ^CY^	34.5 ± 1.4 ^CY^	36.5 ± 1.8	38.3 ± 1.7
Bilirubin (μmol/L)	2.7 ± 0.1	2.3 ± 0.2	2.8 ± 0.1	2.8 ± 0.2	2.6 ± 0.2
Amylase (U/L)	969.0 ± 57.0	748.2 ± 51.8	831.5 ± 66.5	786.5 ± 94.2	822.0 ± 32.3
Lipase (U/L)	21.2 ± 0.6	18.2 ± 0.6	21.5 ± 2.5	25.0 ± 4.9	17.5 ± 0.9
Osteocalcin (ng/mL)	21.3 ± 1.4	8.6 ± 0.5 ^CY^	7.1 ± 0.7 ^CY^	8.1 ± 1.1 ^CY^	6.2 ± 0.6 ^CY^
Alkaline phosphatase (U/L)	201.1 ± 26.3	137.5 ± 17.7	145.6 ± 26.4	248.9 ± 45.4	178.3 ± 14.92
Calcium (mmol/L)	3.0 ± 0.1	2.9 ± 0.1	2.9 ± 0.1	3.1 ± 0.1	3.0 ± 0.1
Phosphorus (mmol/L)	3.5 ± 0.1	2.7 ± 0.2 ^CY^	2.6 ± 0.2 ^CY^	2.6 ± 0.3 ^CY^	2.7 ± 0.1 ^CY^

Data are presented as mean ± SEM (n = 8/group). Statistical significance was determined by one-way ANOVA followed by Tukey’s test; significant differences were considered at *p* < 0.05 and indicated with respect to the corresponding experimental groups (CY, C, E, L+E).

**Table 2:** Activities of superoxide dismutase (SOD; U/mg protein), catalase (CAT; U/mg protein), glutathione peroxidase (GSH–Px; U/mg protein), glutathione reductase (GR; U/mg protein), glutathione S–transferase (GST; U/mg protein) and the concentrations of total glutathione (GSH; µmol/g wet mass), sulfhydryl groups (SH; nmol/g wet mass), lipid peroxides (LPO; nmol/mg wet mass), total oxidant status (TOS; µmol H_2_O_2_/g wet mass), total antioxidant status (TAS; µmol Trolox/g wet mass), and oxidative stress index (OSI; AU) in the liver of young adult (CY) and middle-aged (C) control, liraglutide-treated (L), exercise (E), and combined liraglutide plus exercise (L+E) groups of male rats.

	CY	C	L	E	L + E
**SOD**	17.0 ± 1.4	13.6 ± 0.8	20.5 ± 1.9	16.8 ± 1.4	18.7 ± 1.9
**CAT**	156.1 ± 8.6 ^L+E^	167.7 ± 21.4 ^L+E^	199.0 ± 12.8	152.1 ± 13.6 ^L+E^	246.9 ± 22.08 ^CY,C,E^
**GSH–Px**	458.1 ± 32.6 ^C,E^	240.6 ± 43.6 ^CY^	307.5 ± 45.1	264.1 ± 40.71 ^CY^	355.5 ± 14.89
**GR**	13.2 ± 1.0 ^L+E^	17.82 ± 2.7	16.31 ± 1.5	12.7 ± 0.3 ^L+E^	21.4 ± 2.5 ^CY,E^
**GST**	708.7 ± 37.0 ^C^	395.9 ± 15.6 ^CY,L,L+E^	506.7 ± 57.99 ^C^	443.0 ± 24.02	630.4 ± 59.03 ^C^
**GSH**	5728 ± 108 ^C,L,E,L+E^	3122.9 ± 190.3 ^CY^	1487.3 ± 286.4 ^C,CY^	1827.1 ± 311.1 ^CY^	2224.0 ± 326.7 ^CY^
**SH**	3990.1 ± 189.6 ^C^	5522.8 ± 117.7 ^CY^	4706.4 ± 150.5	4158.2 ± 398.9	4469.0 ± 318.2
**LPO**	10.08 ± 0.39	11.39 ± 1.36	3.85 ± 0.11 ^CY,C,E^	7.87 ± 0.79 ^CY,C,L^	4.94 ± 0.41 ^CY,C^
**TOS**	59.8 ± 2.1 ^C^	82.4 ± 3.1 ^CY^	74.8 ± 4.7 ^CY,E^	101.8 ± 2.9 ^C,CY,L^	70.40 ± 3.4 ^CY,E^
**TAS**	961.3 ± 53.6 ^C^	1562.0 ± 153.8 ^CY^	1614.0 ± 92.8 ^CY^	1479.0 ± 152.5 ^CY^	1680 ± 61.4 ^CY^
**OSI**	5.5 ± 0.8	5.9 ± 0.8	4.7 ± 0.5 ^E^	7.1 ± 0.7 ^L,L+E^	4.2 ± 0.3 ^E^

Data are presented as mean ± SEM (n = 8/group). Statistical significance was determined by one-way ANOVA followed by Tukey’s test; significant differences were considered at *p* < 0.05 and indicated with respect to the corresponding experimental groups (CY, C, L, E, L+E).

## Data Availability

The datasets used and/or analyzed during the current study are available from the corresponding author on reasonable request.

## Supplementary Materials

The following supporting information can be downloaded at https://www.mdpi.com/article/10.3390/antiox14121492/s1, Figure S1. Protocol of Power analysis (A), central and non-central distributions (B), and X-Y plot for a range values (C). Figure S2. Intraperitoneal glucose tolerance test curve. Data are presented as Mean ± SEM (n = 8/group). Figure S3. Representative micrographs of the liver tissue stained with hematoxylin and eosin from young adult (CY;A) and middle–aged (C) control, liraglutide–treated (L), exercise (E), and combined liraglutide plus exercise (L+E) group of middle-aged male rats; undetectable GLP-1R immunostaining of the liver of young adult male rat (F). Table S1: Primer sequences for RT–qPCR.
