# Supplementary material for: Synergistic Effect of Liraglutide and Strength–Endurance Exercise Training on Hepatic Oxidative Stress and Lipid Metabolism in Middle-Aged Male Rats
Source: Antioxidants (Basel). 2025 Dec 12;14(12):1492. doi: 10.3390/antiox14121492 (PMC12730097; doi:10.3390/antiox14121492)
Supplement: Supplementary file 1 [file antioxidants-14-01492-s001.zip › Supplementary Figure S1 revised Vlahovic et al. 2025.pdf]

Synergistic Effect of Liraglutide and Strength-Endurance Exercise Training on Hepatic Oxidative Stress and Lipid Metabolism in Middle-Aged Male Rats

Dragana Vlahović[a], Svetlana Trifunović[a], Slavica Borković–Mitić[a], Sladjan Pavlović[a], Ivona Gizdović[a], Dieter Lütjohann[b], Branko Filipović[a], Ljiljana Marina[c], Branka Šošić–Jurjević[a]

[a] Institute for Biological Research "Siniša Stanković"—National Institute of the Republic of Serbia, University of Belgrade, Bulevar despota Stefana 142, 11108 Belgrade, Serbia

[b] Institute of Clinical Chemistry and Clinical Pharmacology, University Hospital Bonn, Venusberg–Campus 1, 53127 Bonn, Germany

[c] Center for Infertility and Endocrinology of Gender, University Clinical Center of Serbia, Faculty of Medicine, University of Belgrade, 11000 Belgrade, Serbia

A

F tests - ANOVA: Fixed effects, omnibus, one-way

Analysis:	A priori: Compute required sample size		
Input:	Effect size f	=	0.75
	α err prob	=	0.05
	Power (1-β err prob)	=	0.95
	Number of groups	=	5
Output:	Noncentrality parameter λ	=	22.5000000
	Critical F	=	2.6414652
	Numerator df	=	4
	Denominator df	=	35
	Total sample size	=	40
	Actual power	=	0.9607681

B

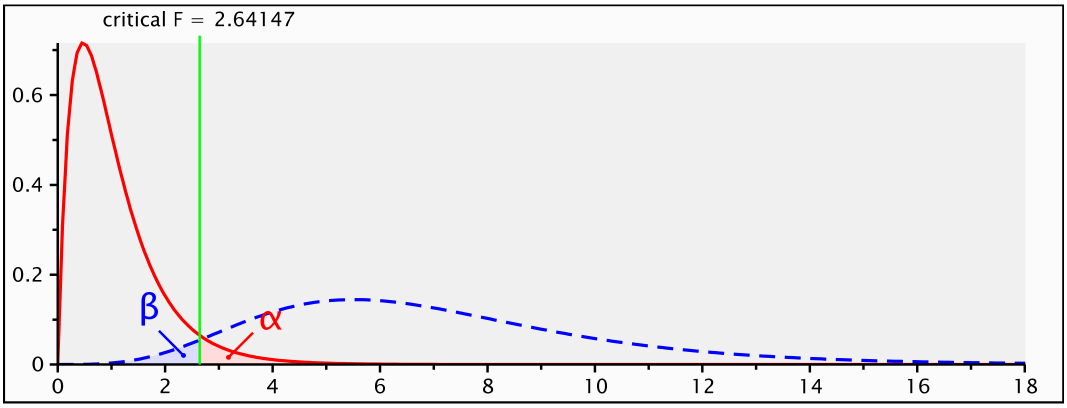

C

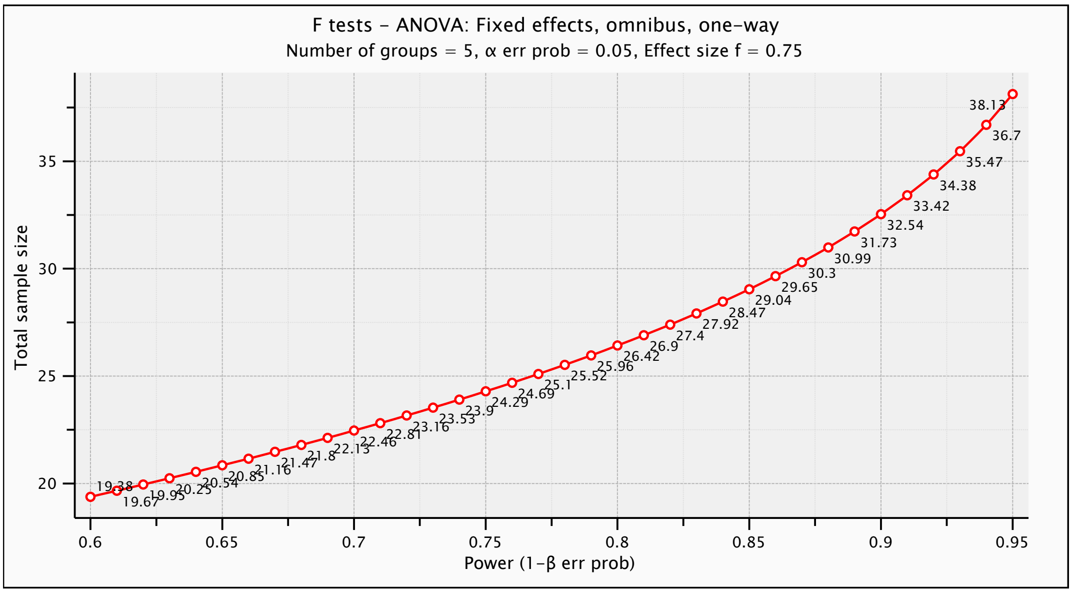

Supplementary Figure S1. Protocol of Power analysis (A), central and non-central distributions (B), and X-Y plot for a range values (C).
