# Supplementary material for: Synergistic Effect of Liraglutide and Strength–Endurance Exercise Training on Hepatic Oxidative Stress and Lipid Metabolism in Middle-Aged Male Rats
Source: Antioxidants (Basel). 2025 Dec 12;14(12):1492. doi: 10.3390/antiox14121492 (PMC12730097; doi:10.3390/antiox14121492)
Supplement: Supplementary file 1 [file antioxidants-14-01492-s001.zip › Supplementary Figure S3 Vlahovic et al 2025.pdf]

Synergistic Effect of Liraglutide and Strength-Endurance Exercise Training on Hepatic Oxidative Stress and Lipid Metabolism in Middle-Aged Male Rats

Dragana Vlahović[a], Svetlana Trifunović[a], Slavica Borković–Mitić[a], Sladjan Pavlović[a], Ivona Gizdović[a], Dieter Lütjohann[b], Branko Filipović[a], Ljiljana Marina[c], Branka Šošić–Jurjević[a]

[a] Institute for Biological Research "Siniša Stanković"—National Institute of the Republic of Serbia, University of Belgrade, Bulevar despota Stefana 142, 11108 Belgrade, Serbia

[b] Institute of Clinical Chemistry and Clinical Pharmacology, University Hospital Bonn, Venusberg–Campus 1, 53127 Bonn, Germany

[c] Center for Infertility and Endocrinology of Gender, University Clinical Center of Serbia, Faculty of Medicine, University of Belgrade, 11000 Belgrade, Serbia

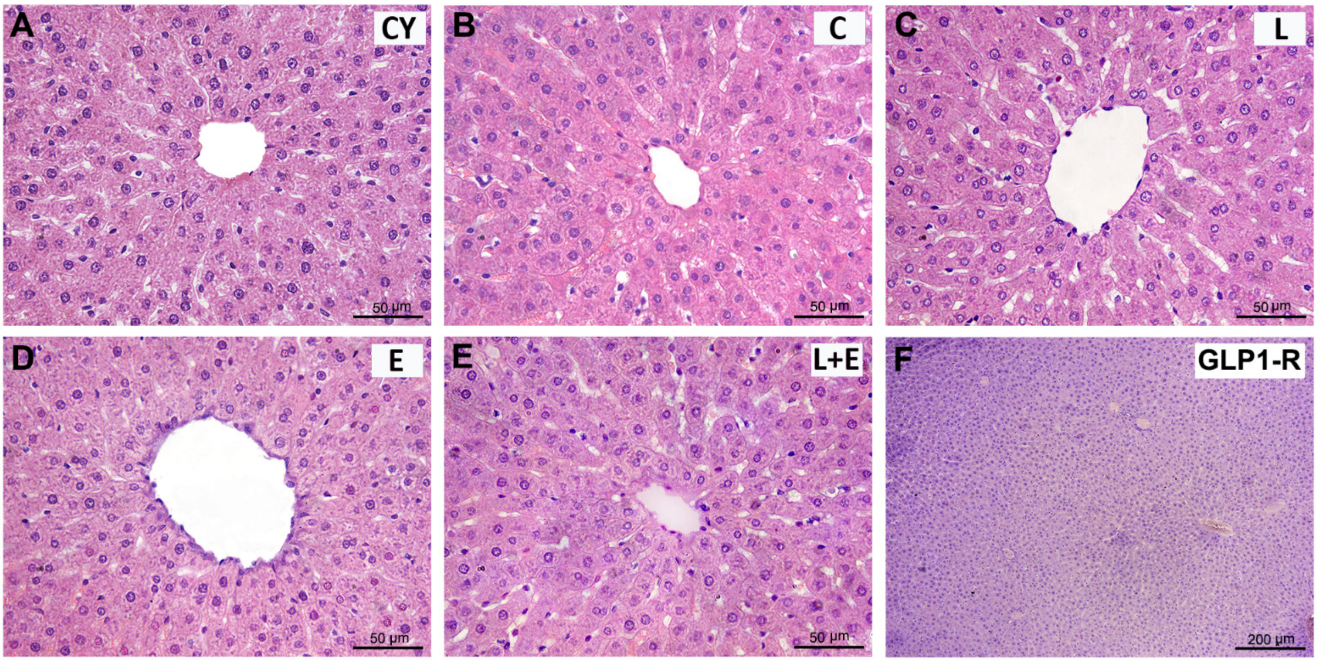

Supplementary Figure S3. Representative micrographs of the liver tissue stained with hematoxylin and eosin from young adult (CY;A) and middle–aged (C) control, liraglutide–treated (L), exercise (E), and combined liraglutide plus exercise (L+E) group of middle-aged male rats; undetectable GLP-1R immunostaining of the liver of young adult male rat (F).
