# Supplementary material for: Synergistic Effect of Liraglutide and Strength–Endurance Exercise Training on Hepatic Oxidative Stress and Lipid Metabolism in Middle-Aged Male Rats
Source: Antioxidants (Basel). 2025 Dec 12;14(12):1492. doi: 10.3390/antiox14121492 (PMC12730097; doi:10.3390/antiox14121492)
Supplement: Supplementary file 1 [file antioxidants-14-01492-s001.zip › Supplementary Table S1 Revised.pdf]

Synergistic Effect of Liraglutide and Strength-Endurance Exercise Training on Hepatic Oxidative Stress and Lipid Metabolism in Middle-Aged Male Rats

Dragana Vlahović[a], Svetlana Trifunović[a], Slavica Borković–Mitić[a], Sladjan Pavlović[a], Ivona Gizdović[a], Dieter Lütjohann[b], Branko Filipović[a], Ljiljana Marina[c], Branka Šošić–Jurjević[a]

[a] Institute for Biological Research "Siniša Stanković"—National Institute of the Republic of Serbia, University of Belgrade, Bulevar despota Stefana 142, 11108 Belgrade, Serbia

[b] Institute of Clinical Chemistry and Clinical Pharmacology, University Hospital Bonn, Venusberg–Campus 1, 53127 Bonn, Germany

[c] Center for Infertility and Endocrinology of Gender, University Clinical Center of Serbia, Faculty of Medicine, University of Belgrade, 11000 Belgrade, Serbia

Supplementary Table S1. Primer sequences for RT – qPCR

Gene	Sequences for forward and reverse primers (5'–3')
Hmgcr	f: TGCTGCTTTGGCTGTATGT<br>r: TGAGCGTGAACAAGAACCA
Cyp7a1	f: *CACCATTCCTGCAACCTTTT*<br>r: *GTACCGGCAGGTCATTCAGT*
Cyp27a1	f: *ATGTGGCCAATCTTCTCTACC*<br>r: *GGGAAGGAAAGTGACATAGAC*
Cyp46a1	f: *TCAGTCATCGTCACGAGTCC*<br>r: *ATACCAGCGCCCATAGTCAC*
Abcg5	f: *GGGTTGCTCATTGGATCTGGA*<br>r: *CCCTTGGGTCATGGAACACA*
Abcg8	f: *CCTTCTACCTTACGGCTGGC*<br>r: *GAGAAGGTGAGGTTGCCGAT*
Fasn	f: TTGCTGGCACTACAGAATCG<br>r: AACAGCCTCAGAGCGACAAT
Scd1	f: TGGTGCTCTTTCCCTGTTTGC<br>r: TGGGCTTTGGAAGGTGGACA
Srebf1 (Srebp–1c)	f: TAGCTCATCAACAACCAAGAC<br>r: TTATGGCACCTGTGTCTGTC
Nr1h3 (Lxra)	f: TCAGCATCTTCTCTGCAGACCGG

	r: TCATTAGCATCCGTGGGAACA
Nr1h2(Lxrb)	f: GGCCGGGAGGACCAGAT<br>r: GCGTCTGGCTGTCTCTAGCAA
Nrf2	f:CACATCCAGACAGACACCAGT<br>r: CTACAAATGGGAATGTCTCTGC
Igf–1	f: GTACCAAAATGAGCGCACCTC<br>r:CCATAGCCTGTGGGCTTGTT
Ar	f: ACCCTCCCATGGCACATTTT<br>r: TTGGTTGGCACACAGCACAG
Thrb	f: GCTAGCCAAGAGGAAGCTGA<br>r: GGGTGCTTGTCCAATGTCTT
Dio1	f: TTTAAGAACAACGTGGACATC<br>r: GTTTACCCTTGTAGCAGATCCT
Hprt	f: GCGCAAGTGTTACGAAGTGG<br>r: AGTGCCCATTTCATTTCGGC
